# Surgical Resection Is Superior to TACE in the Treatment of HCC in a Well Selected Cohort of BCLC-B Elderly Patients—A Retrospective Observational Study

**DOI:** 10.3390/cancers14184422

**Published:** 2022-09-12

**Authors:** Stefania Brozzetti, Chiara D’Alterio, Simone Bini, Jessica Antimi, Bianca Rocco, Alessia Fassari, Pierleone Lucatelli, Piergiorgio Nardis, Michele Di Martino, Giuseppe Maria De Sanctis, Mario Corona, Oreste Bagni, Enrico Cortesi, Mario Bezzi, Carlo Catalano

**Affiliations:** 1 Department of Surgery “Pietro Valdoni”, Policlinico Umberto I., University of Rome-Sapienza, 00161 Rome, Italy; 2 Department of Translational and Precision Medicine, Policlinico Umberto I., University of Rome-Sapienza, 00161 Rome, Italy; 3 Department of Radiological, Oncological and Pathological Sciences, University-Sapienza, 00161 Rome, Italy; 4 Department of Tropical and Infectious Diseases, Policlinico Umberto I., University of Rome-Sapienza, 00161 Rome, Italy; 5 Dipartimento di Medicina Nucleare, Ospedale Santa Maria Goretti, 04100 Latina, Italy

**Keywords:** hepatocellular carcinoma, liver resection, TACE, BCLC-B stage, multidisciplinary tumor board

## Abstract

**Simple Summary:**

Hepatocellular carcinoma (HCC) is the most common primary liver malignancy. Liver transplantation (LT) and surgical resection (SR) are currently the primary treatments with curative intent. Nevertheless, more than two-thirds of patients are elderly and, therefore, excluded from LT; while, according to the Barcelona Clinic Liver Cancer (BCLC) system, SR should only be offered to a small group of patients with early stage HCC. The identification in stage B of an intermediate subgroup of patients that fulfill the criteria for surgery may play an important role in the implementation of potentially curative treatments.

**Abstract:**

Hepatocellular carcinoma (HCC) usually develops in cirrhotic liver, with high recurrence rates. However, considering its increasing detection in non-cirrhotic liver, the choice of treatment assumes particular relevance. This study aimed to investigate outcomes of patients among BCLC stages and enrolled for surgical resection (SR) according to a more complex evaluation, to establish its safety and efficacy. A total of 186 selected HCC patients (median age 73.2 yrs), submitted to SR between January 2005 and January 2021, were retrospectively analyzed. Of which, 166 were staged 0, A, B according to the BCLC system, while 20 with a single large tumor (>5 cm) were classified as stage AB. No perioperative mortality was recorded; complications occurred in 48 (25.80%) patients, and all but two were Clavien–Dindo grade I–II. Median follow-up was 9.2 years. Subsequently, 162 recurrent patients (87,1%) were selected for new treatments. Comparable overall survival rates (OS) were observed at 1, 3, 5, and 10 years in 0, A, B and AB stages (*p* = 0.2). Eventually, the BCLC-B group was matched to 40 BCLC-B patients treated (2015-2021) with TACE. Significant differences in baseline characteristics (*p* <0.0001) and in OS were observed at 1 and 3 years (*p* <0.0001); a significant difference was also observed in oncological outcomes, in terms of the absence, residual, or relapse of disease (*p* <0.05). Surgery might be a valid treatment in HCC for patients affected by chronic liver disease in a condition of compensation, up to BCLC-B stage. Surgical indication for liver resection in case of HCC should be extensively revised.

## 1. Introduction

Hepatocellular carcinoma (HCC) is the most common primary liver malignancy. HCC incidence is growing among the general population, especially in elderly patients. Liver cancer ranks sixth for worldwide incidence and third for worldwide mortality. In Europe it ranks 14th for incidence and eighth for mortality [1,2]. HCC incidence is heterogeneous because of the distribution of its main risk factors: hepatitis B, hepatitis C, alcoholic hepatitis, non-alcoholic fatty liver disease (NAFLD), and steatohepatitis (NASH) [3,4]. Chronic liver disease is the main background in which HCC arises (70–90% of all patients) [5]. Although it usually develops in cirrhotic liver, 10–20% of cases involve patients not yet cirrhotic, with an increasing incidence in the later years [4]. Moreover, 18–22% of HCC patients are older than 75, thanks to medical advancements [3,6] and this rate is expected to become even higher over time [2]. Age is not a good outcome predictor, treatment of older adults must take into account multiple issues related to the condition of ageing itself. First of all, frailty should be considered. Fit patients may indeed tolerate radical and invasive approaches, while unfit patients may not [7]. Surgical resection is the only treatment modality that ensures complete tumor removal and offers the best chance of long-term survival for patients with resectable hepatocellular carcinoma (HCC) [8,9,10,11,12]. Hepatic resection includes major (left hepatectomy, right hepatectomy, trisectionectomy), minor (≤2 Couinaud segments), and wedge resections (non-anatomical resection), with different complexities, technical difficulties, and rates of morbidity and mortality [13]. Among the staging systems available, the Barcelona Clinic Liver Cancer (BCLC) system has been used widely in Western countries [14]. It represented an important step, in order to define prognosis and a therapeutic approach. According to BCLC, tumor burden, degree of liver dysfunction, and the patient’s general condition are the main factors that determine the prognosis and choice of treatment. Several refinements of the BCLC algorithm have been performed in its 20-year history [14]; BCLC stage A has been substantially reclassified adding one subclassification and multiple therapeutic indications; these changes were suggested on the basis of the improvements in evidence arising from HCC management [8,15]. Recently, the 2022 BCLC has recognized liver transplantation (LT) as one of main objectives, widening the indications to a subgroup of BCLC-B patients, in case of successful downstaging by TACE [16]. Conversely, resection has only been recommended for early solitary HCC and, since the official update of 2018, for large HCC when technically feasible; even if with less certainty, due to the lack of robust data [8]. The best choice of treatment in patients with an intermediate state (B) without option for LT is TACE. According to the improvements and evidence arising in HCC management, an extension of resection criteria has recently been advocated, acknowledging that selected BCLC-B patients may benefit more from surgery rather than other locoregional therapies. Thus, the treatment of multinodular HCC is still being debated [17,18,19,20,21,22,23,24,25,26].

The aim of this study was to evaluate the safety and efficacy of surgical resection (SR) in BCLC stage B patients versus the standard treatment according to BCLC guidelines.

## 2. Materials and Methods

Informed consent authorizing the storage and use of all relevant data for research purposes was obtained from all individual patients at the time of enrollment. No further authorization was required from our institutional Ethics Committee, since the study is a retrospective analysis of prospectively collected data, and only de-identified data were analyzed. The informed consent is a written consent signed by the patient.

Data of 186 HCC patients undergoing surgery (aged 65–90 years), over a 16-year period (2005–2021), were prospectively collected in a digital database and analyzed retrospectively. Information regarding clinical data: sex, age, comorbidities, and etiology of hepatopathy/cirrhosis (HBV, HCV, NAFLD, NASH, ASH) were collected.

HCC was staged through imaging techniques, ultrasound (US), computed tomography (CT), and magnetic resonance imaging (MRI), and through laboratory serum α-fetoprotein (AFP) levels.

Health status was evaluated using multidimensional geriatric assessment (MGA score) [7]. Anesthesiological risk was assessed through the American Society of Anesthesiologists (ASA) scale [27]. Surgical risk, predicting operative outcomes, was established according to a score that has been used for 20 years in our Institution, based on age; health status and functional reserve; type of surgery (major, intermediate, minor); and expected postoperative outcomes [28]. Patients with frailty, ASA IV, MGA class 3 and surgical risk score ≥15 were considered unsuitable for any surgical treatment. Liver status was assessed according to the Child–Turcotte–Pugh score (CTP) [29]; the model for end-stage liver disease (MELD) score, the liver stiffness scales (METAVIR scale, Fibroscan values, Fib-4 value) and steatosis (NAS) score [30,31,32,33]. Platelet count, splenomegaly, varices, and serum AFP levels were also considered. Child–Pugh score >8 and Meld >12 were considered absolute contraindications to surgery, as were bilirubin levels >3 mg/dL, associated with PT <50%, INR >1.7. In major resections, the functional reserve of remnant liver was predicted from the indocyanine green retention rate at 15 min (by LiMON^®^, impulse Medical System, Munich, Germany). [28,34]. Future remnant liver was calculated using volumetric CT: an estimated remnant liver volume of ≥35% was a criterion for surgical enrolment.

**Figure 1:**
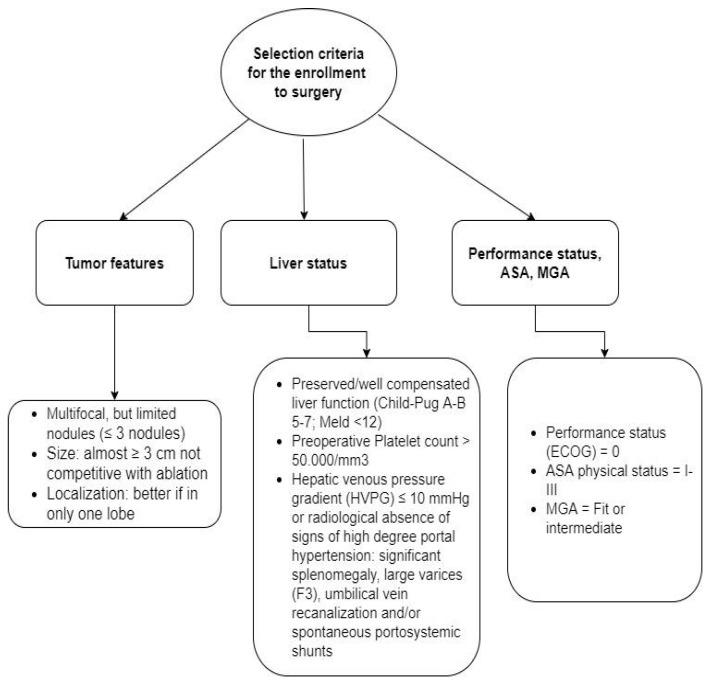
Selection criteria adopted for enrollment to surgery of BCLC-B patients.

According to clinical practice guidelines (BCLC) for the management of HCC by the European Association for the Study of the Liver (EASL2018) [8], patients were classified as stage 0, A and B. However, patients with a single large tumor (>5 cm), since there was not enough data supporting a strong recommendation to surgery until 2018, were classified as stage AB and independently assessed, to better stratify the survival benefit of treatments [12,26]. Four groups were identified: nine patients staged as BCLC 0, 99 as BCLC A, 58 as BCLC B, and 20 patients as stage AB. Therapeutic indication was discussed in a multidisciplinary tumor board made up of hepato-biliary surgeons, interventional radiologists, gastroenterologists, hepatologists, and oncologists, according to multiple prognostic variables, such as tumor features, liver function, and performance status, considering the availability of several evidence-based treatments. In all cases, the treatment decision was hierarchically dictated by the efficacy of each therapy, according to evidence endorsed by the clinical practice of expert centers [19]. Selection criteria adopted for surgical enrollment of BCLC-B patients are shown in Figure 1. Each criterion regarding tumor feature, liver status, and patient’s performance status had to be satisfied to make patients eligible for surgery.

Type of resection, and short and long-term outcomes were registered. Surgical complications were classified according to the Clavien–Dindo classification [35].

All surgical interventions were carried out through an open approach. The Pringle maneuver (inflow occlusion) was always ready-to-perform, but was only completed if risk of excessive blood loss was concrete. Intraoperative US was always performed for a better evaluation of nodules, as a topographic guide to resections and eventual intraoperative radiofrequency ablation. Macroscopic and microscopic surgical margins were determined. SR was histologically defined as R0 (lack of tumor invasion in resection margins) or as R1 (microscopic residual tumor). Three- and six-month follow-ups were performed through US and AFP level evaluation in all patients; CT or MRI were requested routinely at the first year and after every 18–24 months, according to the underlying liver status or in case of a suspected recurrence. Intrahepatic recurrence was defined as the development of de novo tumors (local recurrence or disseminated disease); marginal recurrence was defined as recurrences located along resection margins. One month CT was reserved only for patients who received intraoperative RFA. At each recurrence presentation, treatment was always agreed in a multidisciplinary discussion. Age, general clinical conditions, size, and location of lesions were considered, in order to guarantee best clinical practice. At 1-, 3-, 5-, 10-year follow-ups, patients still alive were assessed for quality of life (QoL) through a WHOQOL-BREF questionnaire [36].

The primary endpoints of this study included perioperative outcomes (up to 90 days after surgery) and overall survival (OS). Secondary endpoints included length of hospital stay (in days), recurrence rates, treatments at recurrence, and quality of life (QoL). The last endpoint was to detect differences in terms of OS and oncological outcomes, comparing SR to TACE in staged B patients.

Clinical data of 40 patients, treated with standard treatment for intermediate stage HCC, in the period January 2005–January 2021, were collected. All TACE indications were discussed at the same multidisciplinary tumor board. Patients presenting with Child–Pugh >B8, portal vein thrombosis (defined as the complete or partial obstruction of blood flow, due to the presence of a chronic, acute, or neoplastic thrombus), extrahepatic secondary lesions, high-flow arterioportal or arteriovenous shunts, platelet count <50,000, or bilirubin level  >3 mg/dL were not considered suitable for the procedure. Patients underwent catheter-based treatment with drug eluted microspheres with a standard micro-catheter (DEM-TACE). The embolization protocol used for DEM-TACE was highly standardized in January 2015. The protocol consisted of a sequential embolization, starting with 100 ± 25 μm PEG microspheres, immediately followed by a second embolization with 200 ± 50 μm PEG microspheres when needed [37]. The number of TACE procedures was decided by the response of tumors to TACE.

In patients submitted to TACE, imaging follow-up was performed at 1 and 3 months and after every 6 months, using either contrast enhanced multi-detector CT or contrast enhanced MRI with hepatobiliary contrast agents. Tumor response was assessed according to mRECIST criteria by a radiologist with  >20 years of experience in CT/MR body imaging, as follows: complete response (CR) was considered as disappearance of any intra-tumoral arterial enhancement in all target lesions; partial response (PR) as a decrease  > 30% in the sum of diameters of viable target lesions (taking as a reference the baseline sum of the diameters of target lesions); stable disease (SD) as any cases that did not qualify for either PR or progressive disease (PD); and PD as an increase of at least 20% in the sum of the diameters of viable target lesions (taking as a reference the smallest sum of the diameters of viable target lesions recorded since treatment started) [38]. If the tumors had a partial response, stable disease, or progressive disease, TACE could be continued, except for the patients with severely impaired liver function.

### Statistical Analysis

Qualitative data were described by frequency and percentage. Quantitative data were described by the median and interquartile range (IQR) or mean and range. Data were analyzed with a Chi-square test, as well as Student’s paired and unpaired t-tests. Actuarial relative survival was described using Kaplan–Meier analysis. A log-rank test was used to compare continuous variables and was expressed using Kaplan–Meier curves. The homogeneity of the different groups to be compared was tested using a chi-square test. Statistical significance was set at *p* ≤ 0.05. Statistical analysis was carried out using the R software (version 3.6.1, Ontario, Canada). Cases (BCLC-B surgical patients) and controls (BCLC-B TACE patients) were matched by age and sex, using propensity score.

## 3. Results

### 3.1. Surgical Resection

#### 3.1.1. Clinical-General Characteristics of Patients

The surgical cohort was composed of 125 males and 61 females. The median age at the time of surgery was 73.2 (IQR:67-81 years) (Table 1).

A total of 27 patients (14.5%) were affected by HBV-related hepatitis, 131 (70.4%) by HCV infection, and 7 (3.8%) by mixed infection. Meanwhile, 19 patients (10.2%) developed HCC on steatosis/NAFLD. The two last patients presented alcohol-related liver disease. A total of 132 patients (70.97%) presented HCC without cirrhosis (METAVIR F0-F1, F2).

A total of 174 patients (93.55%) had CTP score A (5, 6), 12 patients (6.45%) had CTP score B (7, 8), and no patients had CTP score C. Comorbidities and stratifications of patients according to the ASA classification and MGA score are summarized in Table 1.

Nine patients fell within BCLC stage 0, 99 in stage A, 20 in stage AB, and 58 in stage B. Numbers of resected nodules, and the size and type of resection performed are summarized in Table 2.

A significant difference was observed among the four groups in terms of the type of resection: extended right hepatectomy, right hepatectomy, and left hepatectomy were performed in BCLC-AB and B patients, while just one right hepatectomy was performed in a BCLC-A patient. Minor hepatic resections prevailed in the BCLC-A group; wedge resections were performed in two BCLC-A and in four BCLC-B patients. In our cohort, intraoperative radiofrequency ablation (RFA) was combined with surgery with curative intent (for nodules <3cm) when the number of nodules or their position did not allow a surgical resection sparing the parenchyma.

No cases with a microscopic positive surgical margin (R1) resulted at histological exam.

#### 3.1.2. Perioperative Morbidity and Mortality

Overall, 90-day morbidity was 25.80%: no significant differences emerged among the four groups, as reported in Table 2.

Complications occurred in 48 patients (25.8%), and all but two (1.1%) were graded I and II according to the Clavien-Dindo classification. Major complications (Clavien-Dindo grade III) occurred in one BCLC-A and in one BCLC-B patient, and were represented by bleeding in both. One was treated with transfusion and systemic measures of hemostasis, the other with transfusion and re-laparotomy to stop bleeding. No mortality was recorded at 90 days after surgery.

#### 3.1.3. Overall Survival

In January 2021, three patients in stage 0, 37 patients in stage A, seven patients in stage AB, and 28 patients in stage B were dead (Table 3).

The 3-year overall survival was 100% [IC: 1–1] in class 0, 96.03% [IC: 0.917–0.999] in class A, 95.21% [IC: 0.813–0.946] in class AB, and 97.43% [IC: 0.891–0.992] in class B. The 5-year overall survival was 88.9% [IC: 0.706–1] in class 0, 80.8% [IC: 0.589–0.779] in class A, 78.7% [IC: 0.532–0.755] in class AB, and 67.2% [IC: 0.507–0.718] in class B. There were no statistically significant differences between the OS of the four classes (*p* = 0.2), as reported in Table 3 and Figure S1.

The main cause of death in our cohort was liver disease progression (45.3%); 23 patients (6 BCLC-A, 2 -AB and 15 BCLC-B) died because of tumor progression, and 24% of deaths were related to other causes (Table 3).

### 3.2. Follow-Up and Recurrence

Median follow-up was 9.2 years (IQR: 5–12); however, 21 patients did not reach 3-year follow-up, 48 did not reach 5-year follow-up, and 72 patients of this cohort did not reach a 10-year follow-up. A total of 162 patients presented recurrences (87.09%): six (66.6%) in stage 0, 86 (96.87%) in stage A, 12 (60%) in stage AB, and 58 (100%) in stage B (Table 2). In these patients, 12 resections, 110 RF/MW ablation, 150 TACE, four selective internal radiation therapies (SIRT), and six medical therapies (Sorafenib, immunotherapy, best supportive care) were administered. Several patients developed more than one recurrence and were discussed in a multidisciplinary unit at each presentation. They were submitted to different treatments, both curative and palliative, according to their current performance status, liver function, tumor characteristics, and therapeutic hierarchy concept. Details about recurrences and their treatment are reported in Table 2.

Statistically significant differences were found among the four groups, in terms of the curative and palliative treatments performed at first and second recurrences, while no differences resulted in third recurrence treatments (Table 2). There was no mortality at 90 and 30 days, respectively, for surgery retreatments or interventional radiology.

### 3.3. Quality of Life Analysis

There was a significant difference of QoL at 3- and 10-years follow up (*p* = 0.013, *p* = 0.0028, respectively) (Table S1). No patients showed a very poor quality of life. A small number of long-surviving patients at BCLC stage B had excellent QoL 10 years after surgery (Table S1).

### 3.4. Comparison of Surgery and TACE in BCLC-B Stage

A comparison of baseline characteristics of the 58 BCLC-B patients undergoing surgery and 40 BCLC-B patients submitted to TACE is reported in Table 4.

Patients in the TACE group showed a higher age, significantly higher ASA score (*p* = 0.0008), METAVIR score (*p* < 0.0001), grade of varices (*p* = 0.0007), number of nodules (*p* < 0.00001), and rate of bilobar tumor involvement (*p* < 0.0001) (Table 5). The postoperative outcomes between the two treatments are shown in Table 6. TACE reported fewer complications and a faster postoperative recovery, which was statistically significant (*p* = 0.043, *p* <0,05) compared with liver resection.

**Figure 2:**
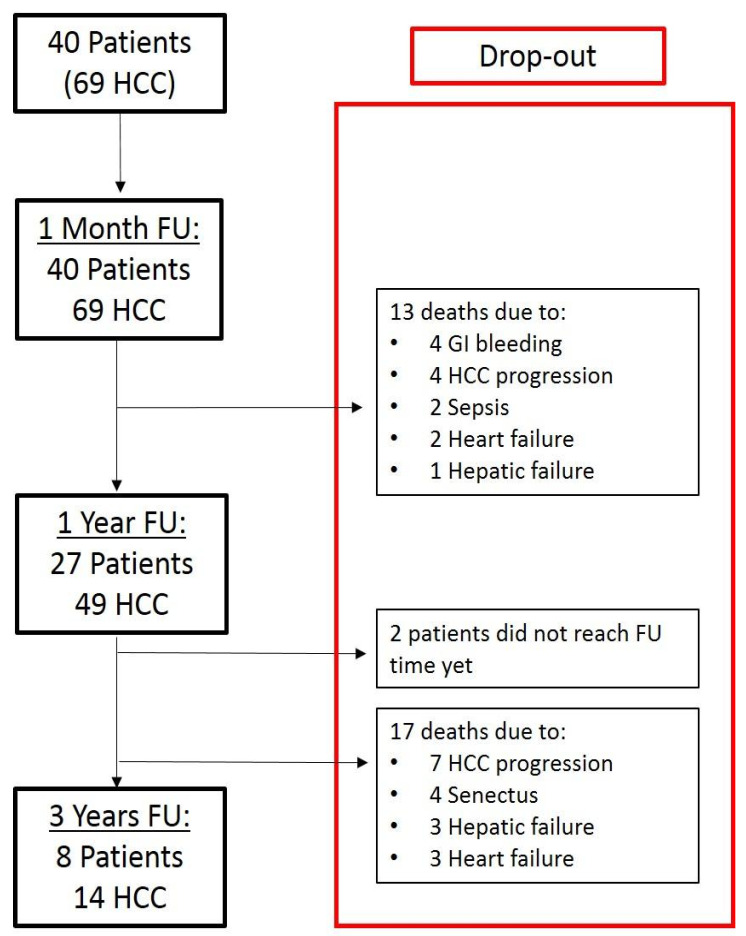
Follow-up in patients submitted to TACE.

Oncological outcomes in surgical and TACE BCLC-B patients are reported in Table S2. At 1-year follow-up all resected patients were alive, while in the TACE group, the 1-year OS was 61.7% [IC 95: 0.5224–0.8296] (*p* < 0.0001) (Table S2, Figure 2).

**Figure 3:**
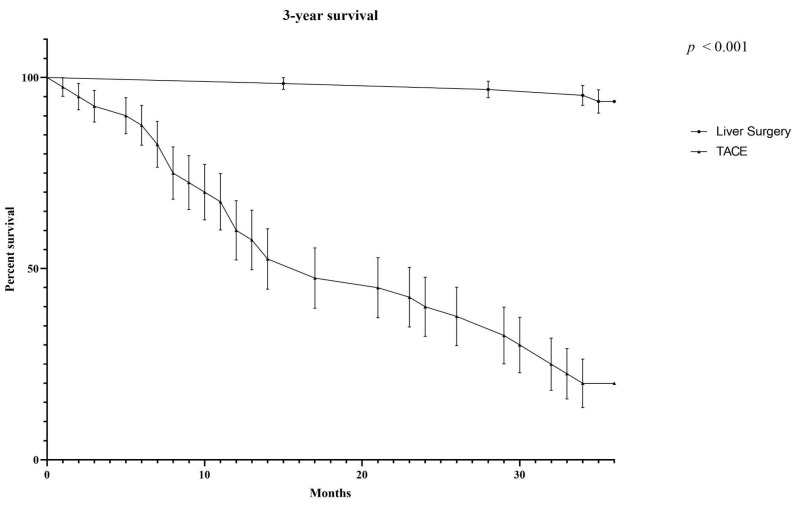
Comparison of OS between the surgical group and the TACE group.

Four TACE patients died due to HCC progression, five due to liver disease complication/hepatic failure, two from sepsis, and one from heart failure. Three-year OS was 97.4% [IC: 0.891–0.992] in the surgical group and 32.3% [IC 95: 0.1762–0.4802] in the TACE group (*p* < 0.0001), as reported in Figure 3.

Nine resected patients did not reach 3-year follow-up; two were dead at 3 years: one due to HCC progression and one from other causes. In the TACE group, two patients did not reach the 3-year follow-up; a further 17 patients died within 3 years: seven due to HCC progression, three from hepatic failure, and seven due to other causes. Liver disease and HCC progression, the main causes of death, had a similar incidence in TACE patients; 26.3% and 28.9%, respectively. However, in HCC, the severity of the underlying cirrhosis may have influenced the overall survival, and it can sometimes be difficult to ascertain whether a given patient died from HCC or cirrhosis.

## 4. Discussion

For a staging system to be effective and widely used, it has to be reliable, reproducible and simple, using data elements that can be obtained as part of standard clinical practice across a wide range of treatment options. The BCLC classification was first published in 1999 [39]. It was derived from a single institution experience, the Barcelona Clinic Liver Cancer group of experts, and approved during the single-topic conference of the European Association for the Study of the Liver (2000 EASL) [40,41]. The BCLC staging system takes into account the size and extent of tumor, liver function, and performance status, with a corresponding treatment schedule and expected survival estimation for each stage. It is considered the standard HCC staging system by the American Association for the Study of Liver Disease (AASLD) [42]. Since its introduction, the BCLC staging system, generated according to the results of randomized controlled studies, has been repeatedly validated and recommended for prognostic prediction and treatment allocation of HCC patients [8,15,43]. Nevertheless, with its repeated updating over time, surgical therapy has been confirmed for a restricted group of early solitary (0, A) HCC patients, and more recently single large tumors (>5 cm), with no vascular invasion or extrahepatic spread, are still considered surgical candidates and allocated as early HCC (BCLC-A). However, this category was estimated to have a worse prognosis than BCLC-A HCC <5 cm, and therefore some authors have suggested designating this subgroup as BCLC-AB or A1 stage [8,12,26]. The outcome of these cases is ill-defined, due to the scarcity of reports. Recent data have shown an improvement in OS with resection compared to TACE [17,18,19,20,21,22,23,24,25,26,44,45].

Ablation or LT represents the only treatment for multinodular BCLC-A patients; while in the intermediate (B) stage (>3 tumors of any size, or 2–3 tumors with a maximal diameter >3 cm, asymptomatic and with no vascular invasion or extrahepatic spread) TACE is the standard of care and the only available option. The indication for hepatic resection is strictly limited in the most recent BCLC staging updates; inversely, several reports in the literature have promoted its wider application [12,17,46,47].

Large and qualified multicentric surveys have shown that surgery is widely applied in current practice among patients with multinodular (stage A and B) and large HCC, providing good short- and long-term results. For these reasons the recommended standard of care of multinodular HCC stage B remains controversial [17,18,19,20,21,22,23,24,25,26,48,49,50].

An attempt to expand the indication of radical treatments for selected patients in certain subgroups was made in the BCLC staging system update in 2022, introducing a number of important changes and additions [16]. However, it still only recommends surgery in very early and early stages for single nodules and according to specific a subclassification, and it still suggests the option of a right to left migration strategy. It introduces the definition of three intermediate stage subgroups. The first BCLC-B subgroup includes patients that could be candidates for LT if they meet the “extended liver transplant criteria”, according to the criteria of the institution. The second subgroup, with well-defined HCC nodules and preserved portal flow, is linked to first-line treatment recommendation TACE. The last, with diffuse, infiltrative, extensive bilobar liver involvement, is linked to systemic treatment, similarly to advanced stage (C). These refinements were aimed only at extending criteria for LT, but none of them focused on the indications for LR. Individualized clinical decision-making is defined by teams responsible for integrating all available data with the individual patient’s medical profile. According to BCLC members and expert authors, despite the encouraging data for select interventions published in reports up to 2021, these are defined as “still too immature to be incorporated into an evidence-based model” [16]. Since these are global issues, we cannot fail to note that this is a setting having an expert team with a prevalence of transplant surgeons and interventional radiologists.

The intermediate stage of HCC is extremely heterogeneous, comprising cases with different factors that strongly affect survival outcomes and treatment response. Therefore, it lacks an internal division able to stratify patients and to also consider LR in the treatment strategy. The vast majority of published studies did not focus on the correlation between tumor characteristics, liver stage, and treatment decisions in stage B patients. It is actually well known that some BCLC-B patients would benefit from surgery, but the hard question is, which of them? In accordance with the claims of other authors [51], tumor features, residual liver function, and patient’s performance status remain critical in the selection of treatments. Multifocal tumors, but with limited nodules requiring no extensive SR, a sufficient liver reserve, and a good patient performance status, identify candidates who may benefit from liver resections.

Our group of surgical patients showed satisfactory OS, with a 5-year OS of 67.2% [IC: 0.507–0.718]. This result is comparable to outcomes of 157 BCLC-B resected patients reported in a multi-institutional analysis of 1010 patients by Tsilimigras et al. [22]. Our results broadly overlap with reports from other study groups: we observed statistically significant higher OS and lower mortality related to liver disease/HCC progression, comparing resected BCLC-B patients to a similar group of TACE patients. Recently, Peng et al., comparing the results of 70 patients undergoing laparoscopic hepatectomy, showed significantly better 1- and 3-years OS than a TACE group; 83% vs. 75%; 56% vs. 15%, respectively (*p* < 0.0001) [52]. It is worth noting that the 1-, 3-, 5- and 10-years OS of our BCLC-B patients is comparable to that of early (0-A) HCC groups. This could be attributable to a proper selection. Liver tumors in our TACE patients showed more aggressive behavior and worse prognosis: they were larger in size, more often bilobar, with higher number of nodules, more advanced liver disease (F3, F4), and worse general condition compared to the surgical group, which could have contributed to the less favorable treatments offered and outcomes. In our cohort, 56.9% of BCLC-B patients undergoing resection had mild/moderate fibrosis (F0-F2).

HCC epidemiology is rapidly changing, with a global declining prevalence of HBV and HCV infections [53]. Conversely, the increasing prevalence of type 2 diabetes mellitus and obesity in western countries is leading to significantly higher rates of non-alcoholic fatty liver disease (NAFLD) and steatohepatitis [54], two coexisting conditions that can lead to liver insufficiency, liver cirrhosis, and HCC. The latter has a prevalence of 10% in case of long lasting severe NAFLD (hepatic fat fraction >30%) or steatohepatitis [55]. The refinement of BCLC indications for BCLC B patients could be important for these metabolic patients, often showing HCC in the absence of cirrhosis. In addition, some common polymorphisms of genes involved in lipid metabolism seem to be associated with the development of liver steatosis and HCC. Several meta-analyses [56,57,58,59] investigated the risk of HCC development in ft homozygous carriers of the PNPLA3 I148M variant, and the identified relative risk ranged from 1.67 to 2.68.

A careful balance among therapeutic options, dictated by the efficacy, risk factors (liver cirrhosis, albumin-bilirubin score, thrombocytopenia, alpha-fetoprotein level, age, comorbidities, and extension/complexity of SR), and factors that could encourage surgical resection (superficial tumor, appropriate localization, the proximity of two nodules, an acceptable performance status and liver function) should be made before any therapeutic choice.

Other questions raised by the present study include the management and outcomes among elderly patients; indeed, the median age of patients enrolled in this study was 73.2 years. Several reports from the USA, UK, and Japan showed a significant age-specific increase in HCC development among persons over 70 years old [60,61]. From recent studies evaluating the clinical characteristics of elderly patients, it emerged that elders have less liver tissue fibrosis and fewer HCC nodules than younger patients [62,63,64].

The results reported in the present study highlight that restricting treatments based only on age cannot be justified. The comparison between life expectancy at operation and the possible extension of life expectancy by surgery has always been performed according to other specialists, including hepatologists, oncologists, and radiologists. We strongly believe that chronological age is not an absolute contraindication for surgical treatment. Physiological age is a new fundamental concept that could better determine fitness for surgery. Therefore, decision-making should always be taken in a multidisciplinary setting.

Despite significant advances in the treatment of HCC, recurrence rates remain high, particularly in intermediate/advanced stages; thus, long-lasting HCC surveillance is necessary to promptly diagnose and treat new nodules. A lifelong follow-up and well-timed intervention may allow B staged patients to reach a survival of decades, if the new therapy is well considered. Recurrence rate (RR) was one of the secondary endpoints analyzed in this study. In the literature, recurrence rates after radical LR are estimated to range between 40% and 75% at 5 years [65,66]. Our report confirmed these data, with a calculated recurrence risk of 87.1%. R1 resection, microvascular invasion, satellitosis, nodules >5 cm, and serum AFP level >100 ng/mL are independent risk factors for HCC recurrence [66,67,68,69]. Signs of tumor aggressiveness include the recurrence site (near vs far from resection) and recurrence timing after surgery (new nodule appearance less than 2 years from surgery is a sign of aggressiveness) [70]. Tsilimigras et al. demonstrated that RR is higher for patients beyond BCLC criteria, but it is similar between BCLC 0/A and BCLC B/C after the second postoperative year [70]. For this reason, close surveillance in the early postoperative period is mandatory. HCC recurrence treatment has been little investigated. Single nodule recurrence may benefit from a second liver surgical resection. In our cohort, when a recurrence was diagnosed, a patient was re-evaluated in a multidisciplinary unit, and resections, RF/MW ablation, TACE, SIRT, and drug therapies (Sorafenib, immunotherapy, best supportive care) were chosen considering liver function and comorbidities, in addition to tumor site, size, and timing from the previous treatment. Aggressive treatment of recurrence by repeat SR, ablation, and adjuvant therapies, such as TACE, can offer satisfactory overall survival (OS) [64].

Lastly, QoL was evaluated in the four groups. QoL is an essential criterion to evaluate in clinical trials and a major predictor of outcomes; however, it is rarely analyzed when running oncologic RCTs. In the last few years, there has been a growing interest in the assessment of QoL for oncological patients [71,72,73,74]. Several studies evaluated the effects of treatment modalities on health-related QoL in HCC patients [74,75,76]. The choice of optimal treatment should always consider health-related QoL. In order to better evaluate patient’s QoL in surgery, we decided to take some parameters from the multidimensional geriatric assessment (MGA score) [7] used in clinical oncology to assess patient’s QoL and fitness to chemotherapy; these parameters were chosen due to the experience gained in treating geriatric patients in the context of our geriatric unit and with the collaboration of the Italian Geriatric Oncologic Group (GOGI) [77]. In our study, SR for HCC provided a satisfactory postoperative QoL, regardless of the preoperative status.

The present study has several limitations. First it is not a randomized study nor an age-sex-comorbidities-matched case-control study. Patients undergoing TACE were not eligible for surgery, due to comorbidities or a higher grade of liver dysfunction. Moreover, patients submitted to TACE often had one or more HCC nodules in a cirrhotic liver or in conditions of partial liver compensation; this is certainly a bias and could partly explain the significant differences in survival shown by the two groups of patients.

Further studies, possibly multicentric prospective randomized trials in high-volume centers are mandatory, to develop a multiparametric prognostic evaluation and to establish a “tailored” operative management for patients with BCLC-B HCC.

## 5. Conclusions

A perfect and unifying HCC staging system does not exist. The BCLC system, endorsed by the European Association for the Study of Liver (EASL) and the American Association for the Study of Liver Diseases (AASLD), holds the great merit of having introduced an evidence-based approach to the prognostication and the treatment of patients with HCC; however, the treatment allocation remains extremely complex for each stage. Over the years, and with the updated version of the BCLC staging system, important changes have been made to improve the applicability of the score. However, surgery remained restricted to a very limited number of patients, thus being one of the ongoing controversies that surround the BCLC system and encouraging an update of the BCLC therapeutic guidelines. A deeper analysis of the indication to liver resection in the intermediate BCLC stage is still required. The space reserved for resection still seems smaller than its real value. In this study we proved that, in case of careful patient selection, recurrence rates and overall survival are strongly in favor of surgery when compared to TACE. The treatment decision is dictated by the tumor size and localization, liver function, patient age, and performance status. Liver resection may be an appreciable approach to treat selected fit intermediate HCC patients, in terms of safety and long-term outcomes. Some critical aspects of the BCLC staging system today may find solutions in discussions among different dedicated specialists of a multidisciplinary board, and the ultimate decision must be taken, not merely on the basis of a simplified algorithm, but by going through a complex process that requires personal insights and expertise. The final analysis should be left to different dedicated specialists in a multidisciplinary board.

## Figures and Tables

**Table 1:** Baseline characteristics of surgically treated HCC patients.

Variable	n, median (IQR)	%
**Age, years,**	73.2 (67–81)	
*Male*	125	67.2
*Female*	61	32.8
**BMI, Kg/m2, median (IQR)**	26.10 (25.65–26.5)	
**Etiology**		
*HBV*	27	14.5
*HCV*	131	70.4
*HBV + HCV*	7	3.8
*NAFLD/NASH*	19	10.2
*Alcohol*	2	1.1
*Hemocromatosis/Wilson*	0	0
**Comorbidities**		
**Cardiovascular**	34	18%
*Hypertension*	105	56.5
**Pulmonary**	61	32.8
**Renal disease**	1	0.5
**Metabolic disease**		
*Diabetes*	29	15.6
*Metabolic syndrome*	3	1.6
**Malnutrition**	4	2.2
**ASA Score**		
I	44	23.7
II	85	45.7
III	57	30.6
**MGA**		
*Fit*	102	54.8
*Intermediate*	84	45.2
*Frail*	0	0
***Child-Turcotte-Pugh score***		
*A*	174	93.55
*B*	12	6.45
*C*	0	0
***MELD, median (IQR)***	7 (5–8)	
***METAVIR***		
*F0-1*	42	22.6
*F2*	90	48.4
*F3*	30	16.1
*F4*	24	12.9
***Steatosis***	37	19.9
***Platelets,* 10⁹/L, median (Range)**	147 (50–362)	
***AFP*** UI/L, median (IQR)	4.5 (2.8–9.32)	
***Varices %***		
*F0*	76	40.86
*F1*	100	53.76
*F2*	10	5.38
*F3*	0	0

**Table 2:** Tumor features and treatments. Perioperative outcomes. Recurrence treatments.

	Stage 0 (n = 9)	Stage A (n = 99)	Stage AB (n = 20)	Stage B (n = 58)	*p* Value
**Nodules resected/ablated, n (%)**					
1	9 (100)	60 (60.6)	20 (100)	0	
2	0 (0)	30 (30.3)/ 10 * (10.1)	0 (0)	40 (51.3)/ 10 *(12.82)	
3	0 (0)	9 (9.1)/ 5 * (5.05)	0 (0)	18 (23)/ 13 * (16.7)	
**HCC size (cm), median, (Range)**	1.7 (1.3–2)	3.2 (0.8–4.5)	7.5 (5–11)	2.6 (0.8-4.5)	
**Type of resection, n (%)**					*0.00008*
Extended Right Hepatectomy	0 (0)	0 (0)	1 (5)	0 (0)	
Right Hepatectomy	0 (0)	1 (1)	0 (0)	9 (15.51)	
Left hepatectomy	0 (0)	0 (0)	1 (5)	2 (3.45)	
Bi-Segmentectomy	0 (0)	52 (52.5)	17 (85)	26 (44.83)	
Segmentectomy	9 (100)	45 (45.4)	1 (5)	20 (34.48)	
Wedge	0 (0)	2 (2%)	0 (0)	4 (6.9)	
**Morbidity n, (%)**					*0.54*
I-II (Clavien-Dindo)	1 (11.1)	22 (22.2)	3 (15)	17 (29.31)	
III (Clavien-Dindo)	0 (0)	1 (1.01)	0 (0)	1 (1.72)	
Length of hospital stay, mean (range)	6 (5–8)	7 (6–15)	7 (6–10)	8 (6–15)	
ICU length of stay, mean (range)	0.5 (0–1)	1.2 (0–3)	1 (0–1)	1.3 (0–3)	
90-days mortality	0 (0)	0 (0)	0 (0)	(0)	
**I recurrence treatment, n (%)**	6 (66.66)	86 (86.87)	12 (60)	58 (100)	
Curative Treatments	6 (100)	47 (54.65)	11 (91.67)	23 (39.66)	*0.003*
Palliative Treatments	0	39 (45.35)	1 (8.33)	35 (60.34)	
**II recurrence treatments, n (%)**	3 (33.33)	38 (38.4)	6 (30)	24 (41.38)	
Curative	3 (100)	9 (23.7)	4 (66.67)	5 (20.83)	*0.013*
Palliative	0 (0)	29 (76.3)	2 (33.33)	19 (79.17)	
**III recurrence treatments, n (%)**	1 (11.11)	19 (19.2)	0 (0)	9 (15.5)	
Curative	1 (100)	6 (31.6)	0 (0)	2 (22.22)	0.6
Palliative	0 (0)	13(68.4)	0 (0)	7 (77.78)	

* Patients who received RFA combined with surgery.

**Table 3:** Overall survival.

	Stage 0	Stage A	Stage AB	Stage B	*p* Value
**1-yr OS,** **survival %** **(IC 95%)**	100% [IC: 1–1]	100% [IC:1–1]	100% [IC: 1–1]	100% [IC: 1–1]	*0.2*
**3-yrs OS, survival %** **(IC 95%)**	100% [IC: 1–1]	96.03% [IC: 0.917–0.999]	95.21% [IC: 0.813–0.946]	97.43% [IC: 0.891–0.992]	*0.2*
**5-yrs OS, survival %** **(IC 95%)**	88.9% [IC: 0.706–1]	80.8% [IC: 0.589–0.779]	78.7% [IC: 0.532–0.755]	67.2% [IC: 0.507–0.718]	*0.2*
**10.yrs OS, survival %** **(IC 95%)**	66.66% [IC: 0.507–0.718]	62.2% [IC: 0.542–0.753]	58.3% [IC: 0.492–0.723]	50.3% [IC: 0.464–0.690]	*0.2*
**Death, n (%)**	3 (33.33)	37 (37.4)	7 (35)	28 (48.28)	*0.015*
HCC	0(0)	6 (16.2%)	2 (28.57)	15 (53.571)	
Liver disease/Cirrhosis	0 (0)	21 (56.8)	3 (42.86)	10 (35.71)	
Other causes	3 (100)	10 (27)	2 (28.57)	3 (10.71)	

**Table 4:** BCLC-B patients of our cohort vs. a cohort of 40 patients. Baseline characteristics.

Variable	Resected pts n = 58 n, median (IQR) (%)	TACE pts n = 40 n, median (IQR) (%)
**Age, years,**	70 (65–77)	76 (68–83)
*Male*	39 (67.24)	35 (87.5)
*Female*	19 (32.76)	5 (12.5)
**Etiology, n (%)**		
HBV	9 (15.52)	3 (7.5)
HCV	46 (79.31)	8 (20)
HBV + HCV	3 (5.17)	0 (0)
NAFLD/NASH	0 (0)	4 (10)
Alcohol	0 (0)	18 (45)
Hemochromatosis/or Wilson’s disease.	0 (0)	0
Mixed etiology	0 (0)	1 (2.5)
Cryptogenetic	0 (0)	6 (15)
**Comorbidities, n (%)**		
**Cardiovascular**	10 (17.24)	27 (67.5)
Hypertension	32 (55.2)	30 (75)
**Pulmonary**	8 (13.8)	11 (27.5)
**Renal disease**	0 (0)	2 (5)
**Metabolic disease**		
Diabetes	8 (13.8)	14 (35)
Metabolic syndrome	0 (0)	10 (25)
**Malnutrition, n (%)**	0 (0)	6 (15)
**ASA Score, n (%)**		*p = 0.0008*
I	14 (24.1)	0 (0)
II	26 (44.8)	12 (30)
III	18 (32.1)	27 (67.5)
IV	0 (0)	1 (2.5)
**MGA, n (%)**		*p < 0.00001*
Fit	25 (43.1)	0 (0)
Intermediate	33 (56.9)	32 (80)
Frail	0 (0)	8 (20)
***Child-Turcotte-Pugh score, n (%)***		*p < 0.0001*
A	54 (93.1)	19 (47.5)
B	4 (6.9)	21 (52.5)
C	0 (0)	0 (0)
***MELD, median [IQR]***	7 [5–8]	10 [8–13]
***METAVIR, n (%)***		*p < 0.0001*
F0-1	6 (10.34)	0 (0)
F2	27 (46.55)	2 (5)
F3	14 (24.14)	14 (35)
F4	11 (18.97)	24 (60)
***Platelets, 10⁹/L, median (Range)***	147 (50–362)	109 (64–154)
***AFP** UI/mL, median [IQR]*	5.2 [3.3–9.8]	9 [6.1–51]
***Varices %***		*p = 0.0007*
*F0*	23 (39.65)	15 (37.5)
*F1*	31 (53.45)	9 (22.5)
*F2*	4 (6.9)	11 (27.5)
*F3*	0 (0)	5 (12.5)

**Table 5:** Tumor features in BCLC-B patients treated with SR or TACE.

	SR/RF 58 pts, n 134 HCC (%)	TACE 40 pts, n 69 HCC (%)	*p* Value
**Number of nodules n, (%)**	134 target HCC 108/26 *	69 target HCC	*<0.00001*
2	40/10 * (68.97)	11 (27.5)	
3	18/13 * (31.03)	9 (22.5)	
>3	0	20 (50)	
**HCC site**			*<0.00001*
Unilobar	55 (94.83)	18 (45)	
Bilobar	3 (5.17)	22 (55)	
**HCC nodules/pts, n (range)**	2 (2–3)	4 (2–9)	
**HCC, Median size (cm), (Range)**	2.6 (0.8–4.5)	2.4 (0.7–12)	

* Patients who received RFA combined with surgery.

**Table 6:** Early outcomes in BCLC-B patients according to type of treatment.

	Surgical Resection	TACE	*p*
			*<0.05*
Length of hospital stay median (range)	8 (6–15)	3 (3–15)	
ICU length of stay, median (range)	1 (0–3)	0 (0)	
90-days mortality	0 (0)	0 (0)	
Perioperative adverse events, n (%)	18 (31)	7 (17.5)	*0.043*
Type of adverse events			
			*0.018*
Ascites n. Volume (cc)	9 (200–400)	0	
Liver failure	0	0	
Bile leak	0	0	
Abdominal collection	0	0	
Bleeding	1	0	
Pleural effusion	7	1	
Wound infection	0	0	
Portal thrombosis	0	0	
Thromboembolic events	0	0	
Fever	0	4	
Other	1	2	

## Data Availability

Available at request from the corresponding author.

## Supplementary Materials

The following supporting information can be downloaded at: https://www.mdpi.com/article/10.3390/cancers14184422/s1, Figure S1: Kaplan-Meyer curves for patients submitted to surgical resection; Table S1: Quality of life analysis of patients submitted to surgery; Table S2: Oncological outcomes in surgical and TACE BCLC-B patients.
