# Supplementary material for: Surgical Resection Is Superior to TACE in the Treatment of HCC in a Well Selected Cohort of BCLC-B Elderly Patients—A Retrospective Observational Study
Source: Cancers (Basel). 2022 Sep 12;14(18):4422. doi: 10.3390/cancers14184422 (PMC9496726; doi:10.3390/cancers14184422)
Supplement: Supplementary file 1 [file cancers-14-04422-s001.zip › cancers-1914143-supplementary.pdf]

Figure S1 – Kaplan-Meyer curves for patients submitted to surgical resection

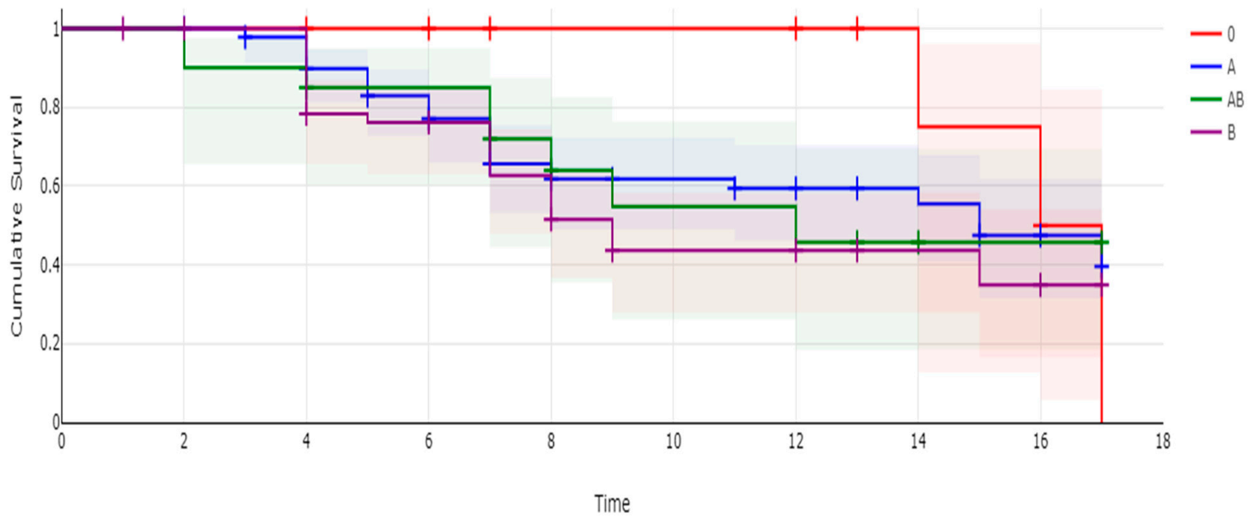

Legend – Survival analysis using the Kaplan-Meyer method for patients submitted to surgery divided by BCLC stage (0,A,AB or B).

Table S1. Quality of life analysis of patients submitted to surgery

	Stage 0-A	Stage AB	Stage B	*p*
1-year follow-up	108 patients:<br>• 23 excellent<br>• 82 good<br>• 3 fair	20 patients:<br>• 6 excellent<br>• 11 good<br>• 3 fair	58 patients:<br>• 15 excellent<br>• 39 good<br>• 4 fair	*0.14*
3-years follow-up	95 patients:<br>• 16 excellent<br>• 76 good<br>• 3 fair	19 patients:<br>• 4 excellent<br>• 11 good<br>• 4 fair	47 patients:<br>• 14 excellent<br>• 28 good<br>• 5 fair	0.013
5 years follow-up	58 patients:<br>• 16 excellent<br>• 36 good<br>• 3 fair<br>• 3 poor	11 patients:<br>• 2 excellent<br>• 6 good<br>• 2 fair<br>• 1 poor	18 patients:<br>• 8 excellent<br>• 7 good<br>• 1 fair<br>• 2 poor	0.39
10-years follow-up	17 patients:<br>• 17 good	5 patients:<br>• 3 good<br>• 2 fair	7 patients:<br>• 2 excellent<br>• 5 good	0.0028

Legend – Quality of life analysis of patients divided by BCLC stage and years of follow-up using WHOQoL-BREF questionnaire.

Table S2. Oncological outcomes in surgical and TACE BCLC-B patients

**Hist. exam, n nodules (%)**	**108 nodules (100) R0**	**-**	***p value***
**1mth**		**40/40 (100) patients<br>69/69 HCC**	
Complete response (target), n (%)	-	28/69 HCC (40.6)	
Partial response (target), n (%)	-	31/69 HCC (44.9)	
Stability of disease (target), n (%)	-	10/69 HCC (14.5)	
Progressive disease (target), n (%)	-	0/69 HCC (0)	
**1 yr**	**58/58 (100) patients<br>108/108 HCC**	**27/40 (67.5) patients<br>49/69 HCC**	
Complete response (target), n (%)	108/108 (100) HCC	26/49 (53.1) HCC	*< 0.0001*
Partial response (target), n (%)	-	17/49 (34.7) HCC	
Stability of disease (target), n (%)	-	3/49 (6.1) HCC	
Progressive disease (target), n (%)	-	3/49 (6.1) HCC	
Intrahepatic local recurrence (*de novo* nodules), n patients (%)	0 (0)	5/27 (18.5) patients	

Intrahepatic disseminated disease, n patients (%)	0 (0)	2/27 (7.4) patients	
**3yrs**	**47/58 (81) patients 91/108 nodules**	**8/40 (20) patients 14/69 HCC**	*< 0.0001*
Complete response (target), n (%)	91/91 (100) HCC	12/14 (85.7) HCC	
Partial response (target), n (%)	**-**	2/14 (14.3) HCC	
Stability of disease (target), n (%)	**-**	0 HCC	
Progressive disease (target), n (%)	**-**	0 HCC	
Marginal recurrence	0 (0)	**-**	
Intrahepatic local Recurrence (*de novo* nodules), n patients (%)	2/47 (4.3) patients	0/8 (0) patients	
Intrahepatic disseminated disease, n patients (%)	2/47 (4.3) patients	1/8 (12.5) patients	

Legend: Complete response, partial response, stability of disease and progressive disease are defined according to mRECIST criteria in target nodules. * Patients who received RFA combined with surgery. Hist: histologically assessed
